# Cohort Succession in the Timing of Marriage Among the Children of Turkish and Moroccan Immigrants

**DOI:** 10.1007/s10680-022-09616-5

**Published:** 2022-05-25

**Authors:** Gusta G. Wachter, Helga A. G. de Valk

**Affiliations:** Netherlands Interdisciplinary Demographic Institute (NIDI)/KNAW/University of Groningen, The Hague, The Netherlands

**Keywords:** Cohort succession, Diffusion, Second generation, Age at marriage, Change, SDT

## Abstract

In this paper, we introduce cohort succession in the study of marriage behaviour among the children of immigrants. Research among majority populations in developed countries has shown an overall increase in age at first marriage. Yet whether a similar change is occurring across successive cohorts of children of immigrants is unknown but relevant given the growing shares of children of immigrants in developed countries. Using full population register data from the Netherlands, we test the theoretical assumptions of cohort succession with event history models for the timing of first marriage across entire Turkish and Moroccan second-generation birth cohorts. In line with the expectations based on diffusion theories, we find clear evidence that younger birth cohorts postpone marriage. Moreover, the marriage timing of especially the Turkish second generation and Dutch majority population converges across birth cohorts. Our findings call for a more differentiated study of the children of immigrants acknowledging diffusion of new demographic behaviour among these groups.

## Introduction

Existing research on union formation among majority populations has shown that since the 1960s, marriages are being postponed or replaced by (longer periods of) unmarried cohabitation in both the USA and Europe (e.g. Billari & Wilson, 2001; Holland, 2017; Manning et al., 2014; Schoen & Canudas-Romo, 2005; Sobotka & Toulemon, 2008). An important process through which these changes have come about is through cohort succession. The continuous entry of new birth cohorts into young adulthood, the period in which most people enter their first unions, allows for changes in union formation patterns as each succeeding cohort has the opportunity to make different choices compared to their predecessors.

Research on the union formation behaviour of majority populations has recognized the importance of changes in demographic behaviour across cohorts (Billari & Wilson, 2001; Liefbroer & Dourleijn, 2006; Nazio & Blossfeld, 2003). Studies on the union formation behaviour of the children of immigrants, on the other hand, have so far mainly focused on differences *between* this so-called second-generation and first-generation immigrants, or their majority population counterparts (Kleinepier & De Valk, 2016; Brown et al., 2008; Hannemann & Kulu, 2015; Kulu & González-Ferrer, 2014; Pailhé, 2015), and rarely considered differences between cohorts *within* the second generation. Although some studies have controlled for period of birth (Georgiadis & Manning, 2011; Hamel et al., 2012; Huschek et al., 2010; Milewski & Hamel, 2010) or interact birth cohort with being a (first-generation) migrant or not (González-Ferrer et al., 2016; Rahnu et al., 2015), a more detailed study of demographic behaviour across second-generation birth cohorts is so far missing. As noted by Hannemann and Kulu (2015), such an endeavour is, however, needed to better understand union formation patterns of the second generation, also in relation to that of the majority population. This is especially important given that the second generation makes up an increasing share of the population in both North America and Europe and their union formation patterns influence future population structures (Hernandez et al., 2009).

Up to now, the study of different cohorts among the second generation has been hampered not only by their group size and related inclusion in data sets, but also by the overall lack of suitable data (Adserà & Ferrer, 2015). In this paper, we make use of full population longitudinal register data of Statistics Netherlands which include all legal residents of the country. These data provide us with a unique opportunity to test theoretical assumptions on cohort succession in the marriage behaviour of entire birth cohorts, defined as all those born in a specific calendar year, of otherwise small to sample populations.

The first question we aim to answer is whether and how the timing of first marriage changes across second-generation birth cohorts. The second research question in this paper relates to understanding whether marriage timing among the second generation and the majority population converges, and if so how important cohort change is in this process compared to other factors of influence. We focus on early marriage patterns (defined here as marriage before age 26) of the Turkish and Moroccan second generation born between 1980 and 1990. These migrant origin groups are particularly interesting to study for three reasons. First of all, with over 400,000 individuals with either a second-generation Turkish or Moroccan background on a total population of around 17 million they are the two largest non-European second-generation groups in the Netherlands (Statistics Netherlands, 2018). Together they account for almost a quarter of the total second generation in 2018 and as such are substantially larger in size than any other non-European origin groups. (Those of Surinamese and Antillean descent follow in this numerical ranking with around 9 and 4 per cent of the total second generation.) Second, with a median age of around 20 years, Turkish and Moroccan second-generation individuals are mainly concentrated around the ages of entering young adulthood including first unions. The age composition of other groups is less suited for our study with several other origin groups being on average older (e.g. German), thus being beyond their main union formation years or still very young (e.g. Polish and Syrian) and thus still in childhood (Statistics Netherlands, 2018). Third, the Turkish and Moroccan second generation are especially interesting because they still follow a more “standard biography” characterized by early (direct) marriage and with unmarried cohabitation being relatively uncommon compared to the (Dutch) majority population. Marriage is furthermore still more key in the union formation process than was or is the case for, for example, the Surinamese and Antillean second generation (Kleinepier & De Valk, 2016). Postponement of marriage across Turkish and Moroccan second-generation birth cohorts can be reflected by a decline of early marriage among these groups. As such this may be an indicator for convergence of marriage patterns towards that of the majority population—among whom postponement of marriage started already in the 1960s.

Because of the importance of marriage in the union formation processes of the Turkish and Moroccan second generation, this paper focusses on cohort change in marriage. This is the first step in understanding how union formation patterns may change over cohorts. Our study adds to the literature in two ways. First, this paper offers a theoretical contribution by applying diffusion theory to the marriage behaviour of the children of immigrants. We argue that this theoretical framework, which has been frequently used to explain changes in demographic behaviour among majority populations, is particularly suitable to study changes in marriage timing across second-generation birth cohorts as it explains how the introduction of new ideas may diffuse among members of a population (Rogers, 1995). In this way, it offers a framework for understanding how the second generation negotiates between “new” marriage norms from their country of birth and “old” marriage norms from their parents’ country of origin, and how these negotiations might result in behavioural changes across birth cohorts. Second, by comparing second-generation birth cohorts to the same birth cohorts of majority group peers, we are able to identify whether differences in marriage timing between these groups become smaller or larger. This is not just interesting in itself but may just as well have major implications for the structural integration of the second generation in society. Marrying at a young age is, for example, shown to be related to shorter educational careers and lower rates of labour market participation, especially among women with an immigrant background (Billari & Philipov, 2004; Blossfeld & Huinink, 1991; Crul, 2000; Dale et al., 2006; Martín-García et al., 2017). Gaining insight into the changes in the marriage behaviour of the second generation is thus essential to shed light on the implications for these other related life course domains where the studied second-generation groups still hold a more disadvantaged position compared to their majority group peers (Gracia et al., 2016).

## Theoretical Framework

### Changes and Differences in Union Formation

In recent decades, the transition to adulthood has changed in many developed countries, including the Netherlands. Whereas marriage was previously a key transition that took place at a relatively young age, this pattern has become less common as marriage, like other key demographic transitions, is postponed among the majority group. This is reflected in the average age at first marriage in the Netherlands which increased between 1960 and 2017 from 24 to 32 for women and from 27 to 34 for men (Statistics Netherlands, 2017). Whereas there was widespread disapproval of unmarried cohabitation before the 1960s (Kalmijn & Kraaykamp, 2018), it has since become the “new norm” to live with a partner in an unmarried cohabiting union before getting married (Billari & Liefbroer, 2010). These union formation changes that occur in many developed countries (Kuo & Raley, 2016; Perelli-Harris & Lyons-Amos, 2015) are often seen as part of the Second Demographic Transition (SDT) (Van de Kaa, 1987) and have been explained by a general ideational change (Surkyn & Lesthaeghe, 2004). It has been argued that due to increased individualization and secularization, the influence of the family and the community has become less important, allowing young adults to make their own individual decisions regarding union formation and other demographic transitions.

The characterization of the SDT as a universal transition is, however, contested (Coleman, 2004), and marriage patterns have not developed in the same ways across countries (Kalmijn, 2007). In Turkey and Morocco, individualization is not as widespread as it is in the Netherlands, and the influence of the family and the community on union formation is still relatively strong (Kavas & Thornton, 2013). Compared to the Dutch majority population, the first immigrants from Turkey and Morocco, who arrived in the Netherlands in the 1960s and early 1970s to fill labour shortages in low-skilled jobs, typically married at much younger ages. They were joined by their families in the 1970s and 1980s and started having children who were born and raised in the Netherlands, i.e. the second generation. The individuals who make up the Turkish and Moroccan second generation are, therefore, still relatively young; 95% of the Turkish and 97% of the Moroccan second generation are currently younger than 40 years old (Statistics Netherlands, 2018). This implies that many of these individuals are in the ages of experiencing key demographic family transitions, like forming a union and getting married, in which they are potentially influenced by both the norms in their country of birth and their parents’ country of origin.

### Diffusion of New Behaviour

In line with assimilation theories that suggest a gradual convergence of behaviour *between* minority and majority groups over generations, previous research in both the USA and Europe has shown that the age at which members of the second generation enter marriage falls between that of the first generation and the majority population (Baykara-Krumme & Milewski, 2017; Brown et al., 2008; Hamel et al., 2012; Pailhé, 2015). Similar results have been found in the Netherlands, where the Turkish and Moroccan second generation marry at older ages than was the case for the previous generation (their parents), but nonetheless at younger ages than the majority population (De Valk, 2008; Kleinepier & De Valk, 2016).

In this paper, we aim to understand whether there is diversity in postponement of marriage *within* the second generation and in particular across birth cohorts as previously observed among majority populations. In order to do so, we turn to theories on diffusion of innovations and ideas. As defined by Rogers (1995, p. 5), diffusion is “the process by which an innovation is communicated through certain channels over time among members of a social system”. Innovation refers to ideas or behaviours that are perceived as new by a group of individuals and that were either absent or rare in the past. Through a process of diffusion, new ideas and behaviours are gradually adopted and become increasingly popular (Granovetter, 1978). This perspective has been previously applied in demographic studies to explain declining fertility rates and changes in union formation and stability across cohorts (Lesthaeghe & Surkyn, 2012; Liefbroer & Dourleijn, 2006; Vitali & Billari, 2017; Vitali et al., 2015).

### Diffusion of New Behaviour Among the Second Generation

Diffusion theories are, however, also particularly suitable for studying changes in marriage timing across second-generation birth cohorts. Turkish and Moroccan immigrants, i.e. the parents of the second generation, faced “new” attitudes regarding union formation and the timing of marriage in the Netherlands as they differed from those that prevailed in their country of origin and in particular in the rural areas where they originated from. Yet according to diffusion theories, the longer the migrant groups are residing in the country of settlement, the more these ideas and norms spread to and are shared within these migrant groups. The older second-generation birth cohorts were the first to be socialized in both the “old”, more traditional ideas about the timing of marriage from their parents’ country of origin, as well as in the “new” ideas about union formation that prevail in the Netherlands. Thus, they were the first cohorts to negotiate between the two cultures and sets of norms, and to be in a position to adopt these new ideas and behaviours.

The intergenerational transmission of norms and values plays an important role in the family (Vedder et al., 2009), and specifically in cultures that put more emphasis on interdependence, as is the case in Turkey and Morocco (Rooyackers et al., 2014; Kagitcibasi, 2013). It has also been shown that immigrant parents in particular attach more meaning to transmitting their norms and values than majority-population parents because they feel that their values are competing with those of the host society (Nauck, 2001). However, the process of transmission appears to be the same in both populations (De Valk & Liefbroer, 2007). Kalmijn and Kraaykamp (2018) also found that parental influence continues to shape the family values of second-generation Turks and Moroccans. Deviating from family or community norms and expectations may affect the family’s position within the larger community and harm family relations. While this pressure will prevent some second-generation young adults from adopting new behaviours, for others, the relative advantages of doing so will outweigh these potential social risks.

According to diffusion theories, innovation of ideas usually starts among a small group of early adopters. We can expect that for some second-generation young adults of Turkish and Moroccan origin, postponing marriage and family formation is seen as desirable because it offers them opportunities to continue their education or build a career (Crul & Doomernik, 2003; Dale et al., 2006). Earlier studies among the majority group have also found that higher educated individuals are often the “frontrunners” in adopting new behaviours (Garssen et al., 2001; Liefbroer & Dykstra, 2000), as compared to their less educated peers, who tend to have more exposure to information and more geographically dispersed social networks (Rogers, 1995). As more individuals follow these early adopters in postponing marriage, the more visible and the less deviating the behaviour will be. Thus, adopting this behaviour will become more attractive and less costly over time. Following this line of reasoning, we can expect that new second-generation birth cohorts entering young adulthood will build on the behavioural changes made by the cohorts who entered union formation ages before them, potentially causing a demographic transition within the second generation. Previous studies that control for birth cohorts support this idea. For example, Georgiadis and Manning (2011), who studied individuals of South East Indian and Caribbean origin born in the UK, found that those born after the 1970s have lower marriage rates at age 25 than those born before the 1970s. Similarly, Huschek et al. (2010), Milewski and Hamel (2010) and Hamel et al. (2012) found that younger second-generation Turks and Moroccans in Europe enter unions at older ages. However, these previous studies either examined cohorts consisting of five or ten combined birth years, combined several origin groups and/or used survey data with relatively small, selective samples. In our study, we are able to disentangle the processes at play between different birth cohorts of Turkish and Moroccan second-generation young adults whereby birth cohorts consist of all people born in specific calendar years. This allows us to more accurately test the hypothesis that marriage will be postponed across birth cohorts of Turkish and Moroccan second-generation even when other socio-economic characteristics are taken into account (H1).

In case postponement among the second generation takes place as expected, then one could subsequently assume the timing of marriage to become more similar to the majority population across birth cohorts. The postponement of marriage among the Dutch majority population already started several decades ago and the “old” norm of early marriage has become rare. Changes in the marriage timing of the latter are therefore expected to be smaller than across second-generation birth cohorts as the second generation was confronted with the “new” norms and entered the diffusion process at a later point in time leaving ample room for new cohorts to adopt to the new norms regarding union formation. Due to these different rates of change among the second generation and majority population, we expect the timing of (early) marriage of the Turkish and Moroccan second generation to converge to that of the majority population across birth cohorts net of other socio-economic characteristics (H2).

## Methods

### Data

We use unique full population register data from the System of Social Statistical Datasets (SSD), of Statistics Netherlands (Bakker et al., 2014). The SSD combines several registers in which each individual is linked through an anonymous personal ID to the Dutch municipal population registers. Thus, the SSD captures a wide range of background characteristics for every official resident in the country. We include all individuals who were born in the Netherlands between 1980 and 1990 and who have at least one Turkish/Moroccan-born parent (*N* = 52,935 Turkish and *N* = 45,145 Moroccan second generation). This definition follows that used by statistics Netherlands, in which an individual’s origin is determined based on the own and parents’ country of birth. Individual ethnic identification is not included in the population registers and thus cannot be assessed. The vast majority (> 90%) of all young adults of the Turkish and Moroccan second generation in the Netherlands have two foreign-born (Turkish/Moroccan) parents due to low rates of interethnic marriage in the parental generation (Statistics Netherlands, 2018). In the few cases in which the parents were born in different foreign countries, we use the country of the mother to determine the person’s origin. Next to the Turkish and Moroccan second generation, we included a 10 per cent random sample of those from the same cohorts but with two parents born in Netherlands (*N* = 166,671 Dutch background). Around 10% of the Turkish and Moroccan second generation and 4% of those with a Dutch background were no longer registered as residents in the Netherlands at the end of our observation window (31 December 2016). As these individuals most likely emigrated or died, we excluded them from the study population resulting in a total of 47,180 Turkish and 40,624 Moroccan second-generation young adults and 159,514 young adults with a Dutch background, all of whom were between 26 and 36 years old at the end of our observation period. The data provide information on all marriage records of these individuals for the 1995–2016 period. As previous studies have shown that men tend to marry at older ages than women, and that members of the Turkish second generation tend to marry at younger ages than their Moroccan counterparts (Distelbrink & Graaf, 2005; Hamel et al., 2012; De Valk, 2008), all of the analyses are conducted separately by origin group and gender.

### Measures

The age at which a person entered a first marriage is calculated by subtracting the date of birth from the date of marriage. Birth cohorts are included by single birth years (1980–1990, recoded to 0–11, respectively) and added as a categorical variable, with 1980 being the reference category.

In order to account for additional factors that influence the timing of entering a marriage, we include several control variables that were shown to affect this transition in previous studies. We start with variables that are relevant for both the second-generation and the Dutch majority population. First, the literature indicates the importance of education, i.e. individuals who are higher educated or still enrolled in education tend to postpone marriage for longer periods of time (Wiik, 2009). Educational level is measured by the highest level of completed education at the end of our observation period. The four categories are (0) low (including primary education, the lower grades of higher secondary education, and completed pre-vocation secondary education), (1) middle (including vocational secondary education and completed higher secondary education), (2) medium–high (including higher professional education) and (3) high (university). The middle category is used as the reference category. As the highest level of completed education is known as of 2006, there are missing values for a small share of our population (6–7% of the Moroccan and Turkish origin groups and 9% for Dutch origin). Multiple imputation was used to deal with this. The data are imputed 10 times using the chained imputation method in STATA (Raghunathan et al., 2001). Values for the highest level of completed education were predicted using the outcome variable, the control variables and the auxiliary variable gender.^1^ Enrolment in education is a time-varying variable measuring, at each age, whether (1) or not (0) an individual’s main activity was being enrolled in education. As this information was included in the registers from 1999 onward, there were missing values at age 16, 17 and/or 18 for the oldest birth cohorts. Given that in the Netherlands, 16- and 17-year-olds are required to be enrolled in full-time education unless they have already earned basic qualifications to enter the labour market, we can assume these young people were in education. The missing values were imputed accordingly. Any missing values at other ages were imputed using the main activity at the first age at which this information is available. Individuals for whom information at all ages was missing were excluded (less than 0.1%).

Second, whether the mother was employed (= 1) or inactive (= 0) at the time the individual was 15 years old was included as an indicator of modernity of the parental home (Schober & Scott, 2012). Children raised in more modern families might postpone marriage more than those from traditional families where early marriage is more common (Huschek et al., 2010). Missing values (less than 2%) were imputed using the same procedure as the highest level of completed education.^2^

The following variables refer specifically to the second generation and are therefore not included in the analyses including the Dutch majority population. First, the number of foreign-born parents an individual has is expected to influence his or her marriage timing. Based on the literature, it can be assumed that the marriage patterns of members of the second generation will be more similar to those of the majority population if they have one foreign-born parent (= 1) than if both of their parents were born abroad (= 0) (Kleinepier & De Valk, 2016).

Moreover, we expect that members of the second generation who have more ties to co-ethnics, indicated by stays in the parental country of origin and the share of same origin residents in the neighbourhood, marry at younger ages (Fokkema et al., 2012; Vervoort et al., 2011). These individuals may be more exposed to and influenced by marriage patterns that are common in their parents’ country of origin. As an indicator of transnational ties, we include a dummy measuring whether a person lived in his or her parents’ country of origin for at least 8 months (1 = yes, 0 = no). A lagged indicator for the ethnic composition of the neighbourhood where the person lived one year before marriage indicates the percentage of residents who are of the same origin. For those who did not marry, we use the percentage 1 year prior to reaching age 25 (the age after which individuals are censored, see below). Because this variable is much skewed towards lower percentages, we use four categories representing the quartiles of the distribution (0 =  < 3.2%, 1 = 3.2 – 7.8%, 3 = 7.9–15%, 4 =  > 15%). For 2.8% of the second-generation individuals, the composition of the neighbourhood is unknown, likely because they were not registered in the Netherlands one year prior to their marriage or before they turned 25. After excluding these individuals, our final analytical sample of the second generation consists of 46,285 Turkish and 39,031 Moroccan young adults. Information on our research population divided by sex and birth cohort/year is shown in Table 1.

**Table 1:** Study population by sex and birth cohort (*N*)

	1980	1981	1982	1983	1984	1985	1986	1987	1988	1989	1990	Total
*Turkish second generation*												
Men	1861	2004	1883	1884	1869	1903	2025	2273	2619	2929	3079	24,329
Women	1685	1913	1650	1753	1714	1678	1804	2061	2339	2578	2781	21,956
*Moroccan second generation*												
Men	1142	1347	1452	1508	1535	1682	1769	1934	2120	2295	2533	19,317
Women	1245	1375	1492	1572	1639	1736	1807	1952	2117	2312	2467	19,714
*Dutch*												
Men	7512	7161	6901	6813	7179	7328	7580	7538	7586	7593	8137	81,328
Women	7209	6923	6860	6753	6871	6906	7266	7290	7355	7250	7460	78,143

### Analytical Strategy

We first present a set of cumulative failure curves that compare the proportion of married Turkish, Moroccan and Dutch young adults by age and birth cohort. To test our first hypothesis, we subsequently estimate discrete-time logistic regression models with robust standard errors that analyse the transition to a first marriage across Turkish and Moroccan second-generation birth cohorts. Whereas in the cumulative failure curves we follow birth cohorts 1980–1990 at all possible ages until the end of our observation period (31-12-2016), we censor the cohorts at age 26 in our event history analyses. We opted for this because all birth cohorts have reached age 26 by the end of 2016 and can be followed for 25 complete years, thereby providing the best comparison across birth cohorts. In a model including all ages, the marriage rates as observed for the oldest cohorts at age 26 and higher would be extrapolated to more recent cohorts who have not yet reached these ages. This, however, does not have to be the case as it may be that younger birth cohorts catch up with higher marriages rates at older ages. By censoring birth cohorts at age 26, we avoid this proportionality assumption. Given that marriage traditionally takes place at a relatively young age for the Turkish and Moroccan second generation, this time window still allows us to observe postponement. The data are organized in a person period file with 1-year time intervals from age 16 through the age at which individuals first married, or age 25 (censored; coded 0–10). The dependent variable measures, at each age, whether a transition to a first marriage occurred (1) or not (0). A quadratic specification is included to model the hazard function, as the effect of age might not be linear. Finally, to test our second hypothesis, we ran a model including the Dutch majority population. Interactions between ethnic origin and birth cohorts were estimated, and the associated predicted probabilities were plotted to show whether the difference between second generation and majority group varies across cohorts. Since our data contain the full population of Turkish and Moroccan second-generation individuals who meet the criteria explained above and a 10 per cent random sample of the Dutch majority population, standard errors and confidence intervals for each of the estimates are reported rather than significance levels (Bernardi et al., 2017).

## Results

### Descriptive Results

Table 2 shows the descriptive statistics for each of the independent variables for the three origin groups by sex. The descriptive statistics given in the first row of the table show that, overall, women are more likely to be married than men. Moreover, the share of married people is higher in the Turkish second generation than in the Moroccan second generation and even more so compared to the Dutch majority population.

**Table 2:** Descriptive statistics of the full study population (1980–1990 cohorts) by origin and sex (%)

	Turkish second generation		Moroccan second generation		Dutch	
	Men	Women	Men	Women	Men	Women
Married^a^	48.9	65.1	37.0	56.2	24.3	34.1
*Highest level of completed education*^*b*^						
Low	24.4	17.3	25.7	15.8	10.9	13.3
Middle	53.8	54.0	51.5	52.8	41.5	48.3
Medium–high	13.6	18.7	14.4	21.5	29.5	22.8
High	8.2	10.0	8.4	9.9	18.1	15.6
*Enrolled in education*						
Age 16	99.5	99.6	99.7	99.8	98.7	99.4
Age 17	94.9	96.5	95.1	96.7	94.0	96.8
Age 18	68.3	74.2	70.9	77.5	66.8	74.2
Age 19	65.7	76.1	70.1	79.2	61.1	72.6
Age 20	54.2	66.1	57.9	68.6	51.9	61.8
Age 21	43.1	54.5	45.7	55.5	43.5	50.5
Age 22	33.2	43.8	34.9	43.2	36.0	39.0
Age 23	25.4	33.7	25.9	31.7	29.0	28.3
Age 24	18.8	24.7	19.0	23.0	22.3	19.5
Age 25	14.1	17.5	13.0	16.0	15.7	12.2
Employed mother at age 15^b^	29.9	29.2	17.2	17.1	62.3	62.5
*% Same origin in neighbourhood*_*t* − 1_						
< 3.2	23.3	22.9	29.6	27.6		
> = 3,2 < 7,9	25.6	25.6	23.5	23.8		
> = 7,9 < 15	26.7	26.3	22.6	23.4		
> = 15	24.4	25.2	24.4	25.2		
One foreign-born parent	5.3	5.3	6.8	6.6		
Moved to country of origin	1.9	2.5	1.7	1.4		

^a^Before the end of the observation period, ^b^based on imputed data. Proportions may not sum up to 100 due to rounding

**Fig. 1:**
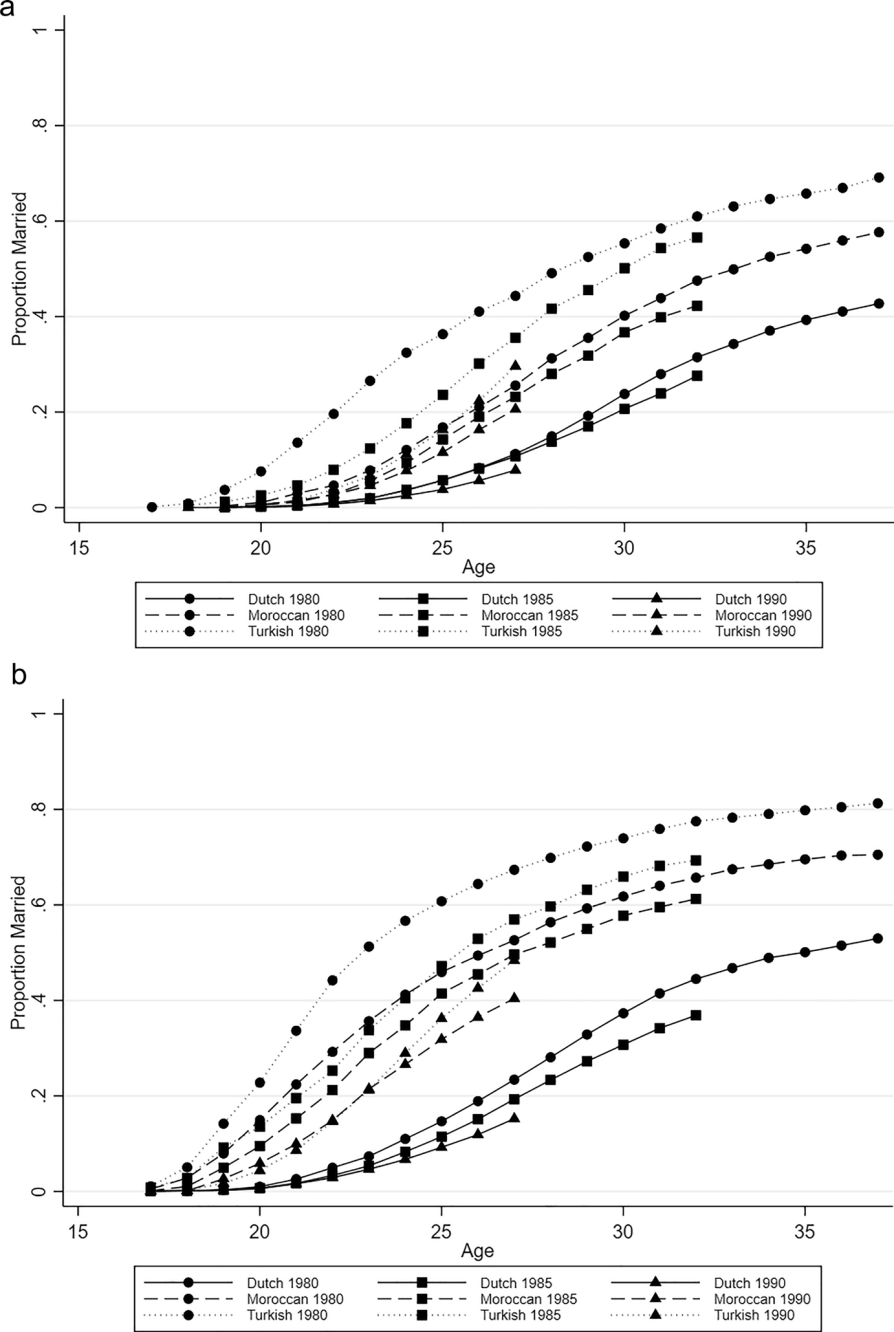
**a** Cumulative failure curves showing the proportion of married men by age, birth cohort and (second-generation) origin, **b** Cumulative failure curves showing the proportion of married women by age, birth cohort and (second-generation) origin

The cumulative failure curves (Fig. 1a, b) show the proportions of married individuals by age and (a selection of) birth cohorts. First, we find that among all groups and both sexes, individuals born in 1980 are more often married at nearly every given age. Second, we observe that although the patterns for the three groups are comparable overall, the Turkish second generation shows the highest marriage rates. Changes are also most pronounced among the Turkish group, as the share of married individuals in this group decreases rapidly over succeeding birth cohorts. Whereas 34% of Turkish women born in 1980 were married at age 21, just 9% of Turkish women born in 1990 were married at the same age (Fig. 1b). For Turkish men, we observe a similar pattern, but a smaller decrease of about 12 per cent points between the 1980 and the 1990 cohort (Fig. 1a). The rate of change across birth cohorts is less pronounced for the Moroccan second generation, and for men in particular. Among the Dutch majority group changes are even smaller. Third, we find that the differences in marriage timing between birth cohorts seem to increase particularly in the early twenties and then become smaller again in the late twenties. For example, if we compare the 1980 cohort and the 1990 cohort of Turkish women, we see a difference of 30 percentage points at age 22, which gradually decreases to a difference of 22 per cent points at age 26 and then becomes much smaller when comparing the 1980 to the 1985 cohort at age 30 (8 per cent points). Fourth, we note that although marriage rates are lower among men than women, a similar pattern of change is observed in both sexes. Finally, we see that the difference between the second generation and the majority population is larger for the older cohorts. When comparing the difference between Turkish second-generation women with majority group women born in 1980 with the difference between those born in 1990 at age 26, we see a decrease of 15 per cent points.

### Explanatory Findings

Table 3 (Turkish) and Table 4 (Moroccans) presents the results from the event history models that examine timing of first marriage across different second-generation birth cohorts. The effect of birth cohorts is first shown without controls for background characteristics in model A and subsequently with the controls for background characteristics in model B. In line with the descriptive findings, we find a clear difference in the timing of entering a first marriage between birth cohorts (model A, Tables 3 and 4). The results from these models largely confirm our expectation that younger birth cohorts of the second generation tend to postpone marriage (H1). For Turkish women, we find a decline in the odds ratio over birth cohorts, which indicates that the younger cohorts of the second generation are postponing marriage. For example, the odds of entering a marriage decrease by 35 per cent for the 1985 birth cohort compared to the 1980 birth cohort. When we compare the 1990 cohort with the 1980 cohort, the difference is even larger: the odds of entering a marriage decrease by more than 50 per cent for those born in 1990. A similar linear pattern is found for Turkish men, with the odds ratio of marriage gradually decreasing with each succeeding birth cohort. Among the Moroccan second generation, the effects are less pronounced. The odds of entering a marriage decrease for the birth cohorts of Moroccan second-generation women as well, but postponement does not take place to the same extent as it does among Turkish second-generation women. This is confirmed by a pooled model including interaction terms between ethnic origin and birth cohorts (detailed results available on request from the first author). For Turkish men and women—and to a lesser degree for Moroccan women—we observe a gradual decrease in the rate of marriage over cohorts. However, for Moroccan second-generation men, we find that the odds ratio changes little across birth cohorts and that there is no clear pattern of delay. As for women, a difference in the effect of especially younger birth cohorts on marriage timing was also found between Turkish and Moroccan second-generation men. Moreover, when we control for background characteristics (model B), we find that birth cohort continues to have an independent effect on the odds of entering a first marriage, with younger birth cohorts clearly delaying marriage more than older birth cohorts (except among Moroccan men).

**Table 3:** Results of discrete-time logistic regression models on the effect of birth cohort on the timing of entering a first marriage among the Turkish second generation

	Turkish							
	Men				Women			
	Model A		Model B		Model A		Model B	
	OR (SE)	95% CI	OR (SE)	95% CI	OR (SE)	95% CI	OR (SE)	95% CI
Age	2.291 (0.056)	2.185–2.403	1.927 (0.050)	1.832–2.027	1.963 (0.029)	1.907–2.021	1.874 (0.029)	1.818–1.983
Age squared	0.956 (0.002)	0.952–0.960	0.964 (0.002)	0.960–0.968	0.954 (0.001)	0.951–0.957	0.954 (0.001)	0.951–0.957
Birth cohort (ref = 1980)								
1981	0.850 (0.048)	0.760–0.951	0.840 (0.048)	0.750–0.940	0.971 (0.048)	0.882–1.069	0.961 (0.048)	0.871–1.060
1982	0.785 (0.046)	0.700–0.880	0.779 (0.046)	0.694–0.874	0.837 (0.043)	0.757–0.925	0.890 (0.047)	0.803–0.987
1983	0.709 (0.041)	0.632–0.794	0.709 (0.042)	0.632–0.796	0.802 (0.040)	0.728–0.885	0.842 (0.043)	0.762–0.932
1984	0.630 (0.037)	0.561–0.707	0.659 (0.039)	0.587–0.740	0.734 (0.036)	0.666–0.809	0.779 (0.040)	0.704–0.862
1985	0.619 (0.036)	0.552–0.694	0.649 (0.038)	0.578–0.729	0.644 (0.032)	0.584–0.711	0.708 (0.037)	0.639–0.784
1986	0.549 (0.032)	0.490–0.616	0.581 (0.034)	0.518–0.653	0.570 (0.028)	0.518–0.628	0.607 (0.031)	0.549–0.672
1987	0.511 (0.030)	0.457–0.573	0.545 (0.032)	0.486–0.611	0.543 (0.026)	0.495–0.596	0.588 (0.029)	0.534–0.648
1988	0.457 (0.026)	0.409–0.511	0.481 (0.028)	0.429–0.538	0.496 (0.023)	0.453–0.544	0.543 (0.026)	0.494–0.597
1989	0.467 (0.026)	0.419–0.520	0.491 (0.027)	0.440–0.547	0.482 (0.022)	0.441–0.527	0.520 (0.025)	0.474–0.572
1990	0.427 (0.024)	0.383–0.475	0.452 (0.025)	0.405–0.505	0.444 (0.020)	0.406–0.486	0.477 (0.023)	0.434–0.523
Employed mother age 15			0.970 (0.027)	0.919–1.025			1.032 (0.024)	0.986–1.079
One foreign-born parent			0.273 (0.026)	0.226–0.330			0.360 (0.023)	0.318–0.407
Moved to country of origin			1.098 (0.093)	0.931–1.296			0.991 (0.067)	0.868–1.133
Educational level (ref = Middle)								
Low			0.730 (0.024)	0.686–0.778			1.058 (0.035)	0.991–1.130
Medium–high			1.042 (0.042)	0.963–1.126			0.756 (0.021)	0.716–0.797
High			0.871 (0.047)	0.784–0.967			0.457 (0.019)	0.421–0.497
Enrolled in education			0.342 (0.013)	0.318–0.368			0.520 (0.013)	0.495–0.547
% Same origin in neighbourhood								
> = 3,2 < 7,9			1.291 (0.050)	1.197–1.392			1.258 (0.039)	1.184–1.337
> = 7,9 < 15			1.405 (0.053)	1.304–1.512			1.298 (0.040)	1.221–1.378
> = 15			1.620 (0.061)	1.503–1.745			1.457 (0.045)	1.371–1.548
Constant	0.003 (0.000)	0.003–0.003	0.007 (0.001)	0.006–0.008	0.018 (0.001)	0.016–0.020	0.028 (0.002)	0.025–0.031
*N* Individuals	24,329		24,329		21,956		21,956	
*N* Person-years	225,609		225,609		178,175		178,175	

*OR* odds ratio; *SE* standard error; 95% *CI* 95 per cent confidence interval

**Table 4:** Results of discrete-time logistic regression models on the effect of birth cohort on the timing of entering a first marriage among the Moroccan second generation

	Moroccan							
	Men				Women			
	Model A		Model B		Model A		Model B	
	OR (SE)	95% CI	OR (SE)	95% CI	OR (SE)	95% CI	OR (SE)	95% CI
Age	3.248 (0.174)	2.923–3.608	2.847 (0.154)	2.561–3.166	2.138 (0.038)	2.064–2.214	2.057 (0.038)	1.985–2.133
Age squared	0.941 (0.004)	0.933–0.949	0.946 (0.004)	0.938–0.954	0.945 (0.002)	0.941–0.948	0.944 (0.002)	0.941–0.948
Birth cohort (ref = 1980)								
1981	0.737 (0.072)	0.609–0.892	0.726 (0.071)	0.600–0.879	0.923 (0.057)	0.818–1.042	0.917 (0.057)	0.811–1.037
1982	0.825 (0.076)	0.689–0.989	0.823 (0.076)	0.687–0.987	0.951 (0.057)	0.846–1.069	0.953 (0.058)	0.846–1.072
1983	0.848 (0.076)	0.711–1.012	0.864 (0.078)	0.723–1.031	0.931 (0.055)	0.830–1.044	0.932 (0.055)	0.830–1.047
1984	0.872 (0.078)	0.732–1.040	0.873 (0.079)	0.732–1.041	0.788 (0.046)	0.702–0.884	0.813 (0.049)	0.723–0.915
1985	0.879 (0.077)	0.740–1.044	0.888 (0.078)	0.747–1.056	0.842 (0.048)	0.752–0.942	0.880 (0.051)	0.786–0.986
1986	0.828 (0.073)	0.697–0.984	0.821 (0.072)	0.691–0.976	0.821 (0.046)	0.735–0.917	0.846 (0.048)	0.756–0.947
1987	0.867 (0.074)	0.733–1.026	0.876 (0.076)	0.740–1.038	0.762 (0.043)	0.682–0.851	0.780 (0.045)	0.697–0.872
1988	0.767 (0.066)	0.648–0.907	0.781 (0.067)	0.659–0.925	0.725 (0.041)	0.649–0.809	0.744 (0.042)	0.666–0.832
1989	0.721 (0.062)	0.609–0.852	0.737 (0.063)	0.623–0.872	0.688 (0.038)	0.617–0.766	0.700 (0.039)	0.627–0.781
1990	0.738 (0.062)	0.626–0.869	0.757 (0.064)	0.642–0.893	0.616 (0.034)	0.553–0.687	0.615 (0.035)	0.551–0.687
Employed mother age 15			0.904 (0.047)	0.817–1.001			0.919 (0.031)	0.860–0.981
One foreign-born parent			0.488 (0.050)	0.400–0.596			0.325 (0.023)	0.284–0.373
Moved to country of origin			1.190 (0.155)	0.922–1.537			1.104 (0.106)	0.915–1.333
Educational level (ref = Middle)								
Low			0.620 (0.030)	0.565–0.681			0.972 (0.035)	0.906–1.043
Medium–high			1.367 (0.068)	1.240–1.506			0.817 (0.024)	0.771–0.866
High			1.160 (0.076)	1.020–1.320			0.625 (0.027)	0.574–0.682
Enrolled in education			0.445 (0.023)	0.403–0.492			0.605 (0.017)	0.573–0.640
% Same origin in neighbourhood								
> = 3,2 < 7,9			1.214 (0.062)	1.097–1.342			1.192 (0.040)	1.117–1.272
> = 7,9 < 15			1.409 (0.071)	1.277–1.555			1.315 (0.043)	1.232–1.403
> = 15			1.392 (0.069)	1.263–1.534			1.334 (0.043)	1.252–1.422
Constant	0.000 (0.000)	0.000–0.000	0.000 (0.000)	0.000–0.001	0.010 (0.001)	0.009–0.011		
*N* individuals	19,317		19,317		19,714		19,714	
*N* Person-Years	186,858		186,858		166,933		166,933	

*OR* odds ratio; *SE* standard error; 95% *CI* 95 per cent confidence interval

The control variables generally have the expected effects. As anticipated, we observe that members of the second generation with one foreign-born parent are more likely than those with two foreign-born parents to postpone marriage, regardless of gender and background.^3^ We also find that for both second-generation groups, being enrolled in education decreases the odds of entering a marriage, and living in a neighbourhood with a higher share of residents of the same origin increases the odds of entering a marriage. Having lived in the parents’ country of origin for a substantial period of time is shown to slightly increase the odds of entering a marriage for both groups. Having an employed mother at age 15 overall only has a very small delaying influence on getting married.

The findings regarding the highest level of completed education do not follow a clear gradient, but instead seem to be dichotomous, and to differ for men and women. Among Turkish and Moroccan women, we see that those who are highly educated are less likely to enter a marriage than those who have a middle level of education, but that the odds of marrying hardly differs between those with a lower and a middle level of education. In other words, there seems to be a dichotomy in the timing of marriage between women who are highly educated and women who are less educated. For Turkish men, the main distinction seems to be between those with the lowest and the highest levels of education, who are both less likely to marry; and those with a middle or medium high level of education, who are more likely to marry. For Moroccan men, we find that, contrary to the other groups, highly educated individuals are more likely to marry at a young age than their less educated counterparts.

The presented models so far have been built on the assumption that the difference between birth cohorts is the same at each age. Additional robustness analyses, testing this proportionality assumption among the second generation, however, show that the effect of birth cohort varies with age except for Moroccan men (Figs. 3, 4, 5, 6, 7 and 8 in the Appendix). Especially for the Turkish second generation, we find the largest differences between birth cohorts in first marriage rates around age 20 (Figs. 5 and 6 in the Appendix). At age 25, the marriage rate is higher for some of the more recent birth cohorts compared to the older ones. For the Dutch majority population, on the other hand, we did not find the difference between 1980 and later cohorts to vary by age (Figs. 7 and 8 in the Appendix). At all ages, younger cohorts were less likely to marry. Similar models were estimated for birth cohorts 1980–1986 until age 30 to examine whether effects remain the same at older ages. These analyses indicate that after age 25, younger cohorts of Turkish second-generation young adults have higher marriage rates than the older cohorts. This holds especially for men and to a lesser extent for women. It seems that younger cohorts of second-generation Turkish thus postpone early marriage yet they “catch up” somewhat at older ages. This was not found for the Moroccan second generation. After adding the control variables to these non-proportional models, the before reported effects remained by and large the same (detailed results available upon request).

**Fig. 2:**
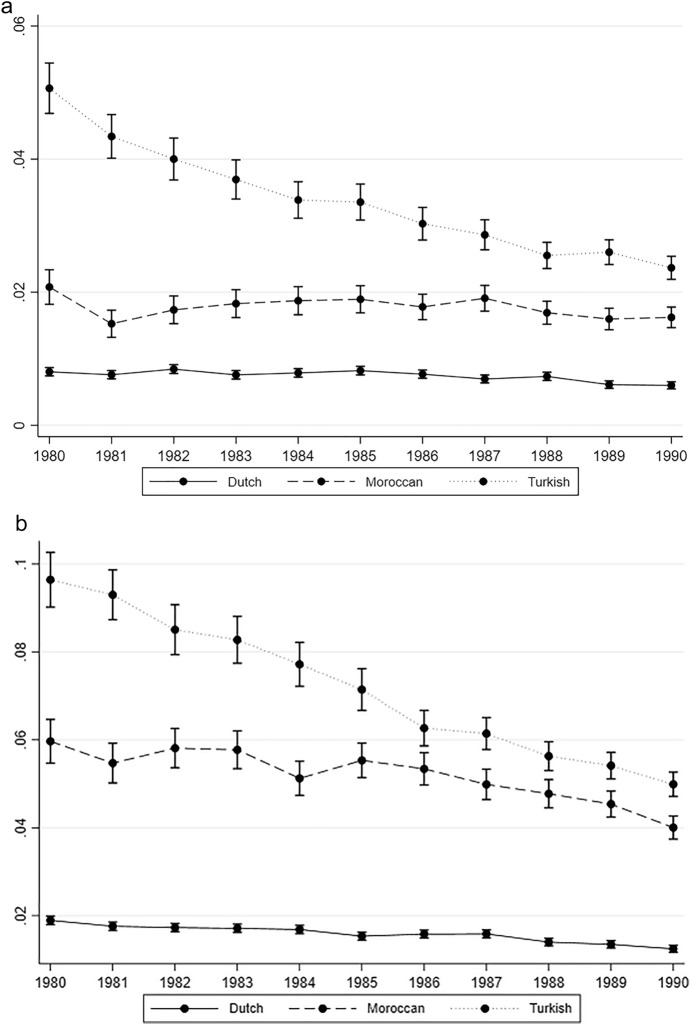
**a** Predicted probabilities of entering marriage showing the interaction between ethnic origin and birth cohort for men, **b** Predicted probabilities of entering marriage showing the interaction between ethnic origin and birth cohort for women

**Fig. 3:**
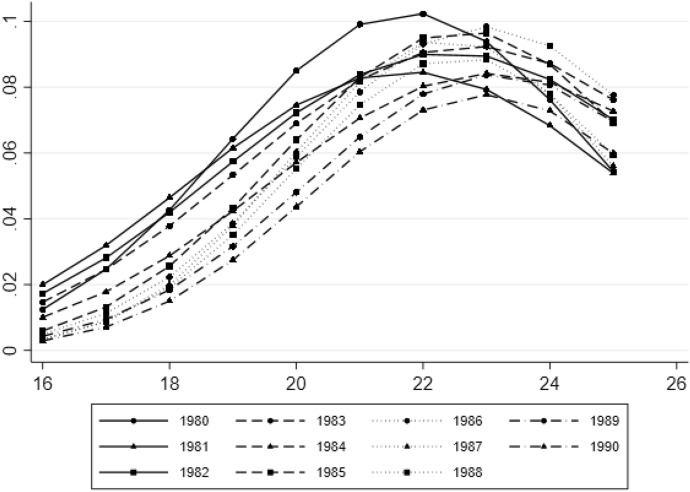
Non-proportional hazards by birth cohort and age, Moroccan second-generation women

**Fig. 4:**
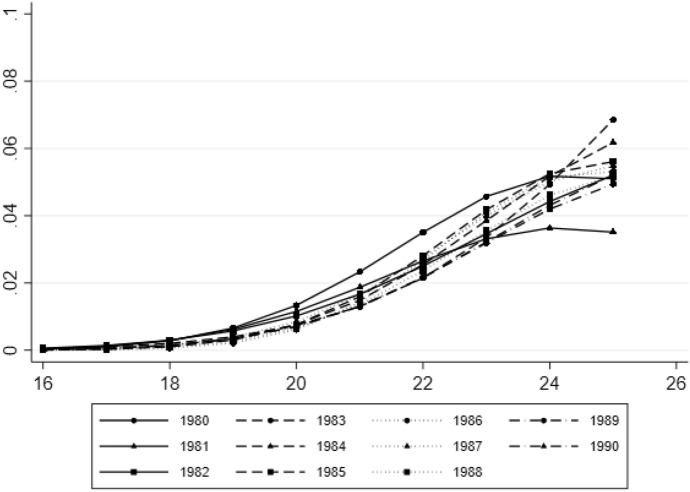
Non-proportional hazards by birth cohort and age, Moroccan second-generation men

**Fig. 5:**
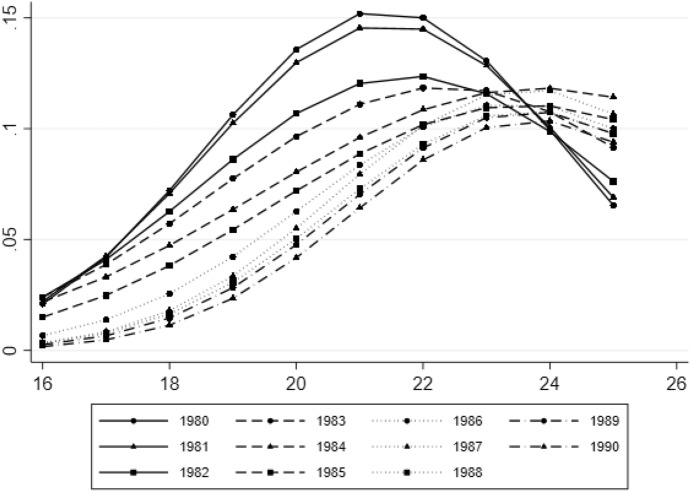
Non-proportional hazards by birth cohort and age, Turkish second-generation women

**Fig. 6:**
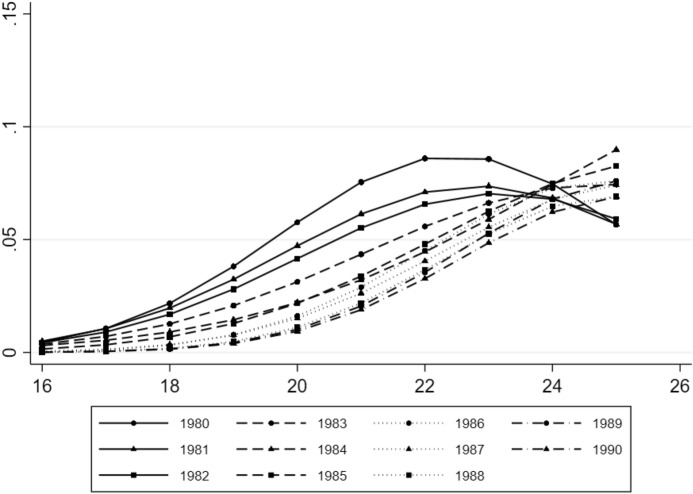
Non-proportional hazards by birth cohort and age, Turkish second-generation men

**Fig. 7:**
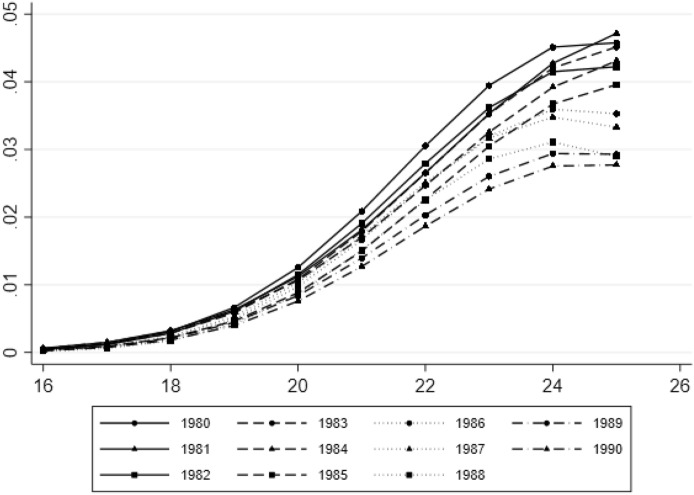
Non-proportional hazards by birth cohort and age, Dutch women

**Fig. 8:**
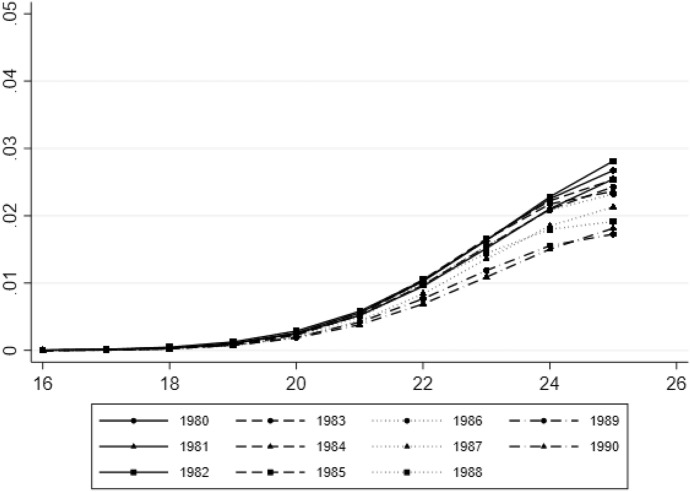
Non-proportional hazards by birth cohort and age, Dutch men

To test our second hypothesis, in which we expect the marriage timing of the second generation to converge to that of the majority population across birth cohorts, we added the Dutch peers to the model and included interaction terms between origin and birth cohort. We estimated the predicted probabilities of entering marriage for the three origin groups across birth cohorts while using average adjusted predictions for the controls (based on Table 5 in the Appendix).^4^ Figure 2a, b shows that the predicted probabilities of marriage for the Turkish group are more similar to that of Dutch majority peers for more recent birth cohorts, thereby indicating the convergence of marriage timing. Whereas the predicted probability for marriage among women of the Turkish second generation born in 1980 is almost 10 per cent (note that this is the average over all person-period years/ages), this is about 2 per cent for the Dutch peers born in the same year. For the 1990 cohort, this difference of 8 per cent points diminished to about 4 per cent points. The Moroccan second generation takes an intermediate position in our analyses on timing of marriage. Older cohorts are already much more similar to their Dutch peers than is the case for the Turkish second generation, especially men. Although the predicted probability is higher for the Moroccan second generation in each birth cohort and decreases, the difference with Dutch peers changes less clearly across birth cohorts than is the case for the Turkish second generation. Convergence of marriage timing is thus much clearer for the Turkish second generation than it is for the Moroccan group. This implies that convergence mainly seems to take place when initial differences in marriage timing are large.

## Conclusion and Discussion

The aim of this study was to examine cohort succession with regard to the timing of marriage among the second generation. Our first research question was whether and how the timing of first marriage changes across second-generation birth cohorts. The second question was whether the marriage timing of the second generation and the majority population converges across birth cohorts. We examined this by taking the Turkish and Moroccan second generation in the Netherlands as a case study and compared them to Dutch majority group peers born in the same years. In line with our expectations, we found that younger birth cohorts of the Turkish and Moroccan second generation postpone entering a first marriage. Even when controlling for background characteristics, clear differences between birth cohorts remained, which suggests that the observed changes in the timing of entering a first marriage are not due to changes in the composition of the second generation. Despite some further marriage postponement among the majority population, timing is changing most rapidly among the Turkish second generation. As such our findings thus suggest that the marriage timing of the Turkish second generation converges to that of the majority population, at least until age 26. No clear evidence was found for the convergence of marriage timing for the Moroccan second generation. This may be explained by the smaller initial differences in marriage timing between the Moroccan second generation and Dutch peers. Especially among men, the difference in first marriage timing is already small for the older cohorts which leaves little room for convergence to take place.

We descriptively followed some cohorts up until their mid-thirties (which was not possible for the youngest cohorts yet). These findings suggest that the differences between birth cohorts in the timing of marriage become smaller from the late twenties onward which indicates that members of the second generation have been postponing marriage, but are not necessarily foregoing marriage altogether. This is further indicated by the results of our non-proportional models showing that the marriage rates of some younger birth cohorts are higher at age 25 and over compared to older cohorts, especially among the Turkish second generation. The observed patterns may be attributable to the fact that marriage is increasingly preceded by a period of unmarried cohabitation, a trend that has delayed the entrance into marriage among majority populations in Northern and Western Europe (Perelli-Harris & Lyons-Amos, 2015). Unmarried cohabitation has so far been relatively uncommon among the Turkish and Moroccan second generation (Huschek et al., 2011). In order to draw more concise conclusions about whether the postponement of marriage coincides with an increase in unmarried cohabitation, future research on cohort succession among the second generation should broaden the types of unions examined. Moreover, as time passes, we will be able to follow second-generation birth cohorts over a longer time period. Interesting in this regard is whether this will imply that the share of unmarried/never married people increases, or whether marriage is just postponed but still taking place in the majority of lives among these origin groups, with most people marrying at some point in (young) adulthood.

Although the findings in this study generally show that younger birth cohorts of the second generation are postponing marriage, we observed differences in the degree of change between the two origin groups. The birth cohort effect was found to be stronger among the Turkish than the Moroccan second generation. One explanation for this difference is that the overall marriage rates were much higher among the older Turkish cohorts, which left more room for change in subsequent cohorts. Second, these different rates of change may also be related to differences between the Turkish and Moroccan communities. Diffusion theory argues, based on homophily principles, that communication between members of a social group is easier when they are more similar to each other. Thus, new ideas are expected to spread more quickly through a relatively homogenous group (Rogers, 1995; Young, 2009). The Turkish community in the Netherlands is characterized by strong social cohesion and collectivism, whereas the Moroccan community is more diverse and individualistic (Crul & Doomernik, 2003). While it may take time for new ideas about the timing of marriage to penetrate the Turkish community, the diffusion process within the community may be rapid once a certain threshold is reached. Testing this assumption requires other type of more in-depth data that we do not have at hand in our study but which would be interesting to pursue in future work.

For both second-generation groups, we observed that the gender differences are more pronounced when it comes to absolute marriage rates than with regard to changes across birth cohorts. In line with earlier findings (e.g. Distelbrink & Graaf, 2005), Moroccan men deviated most from this pattern as they are the ones who are the least likely to marry at a young age. Previous studies have suggested that compared to Moroccan girls, Moroccan boys have (and take advantage of having) more freedom when it comes to exploring relationships, and that their behaviour in this domain is less relevant for the honour of the family. This implies that Moroccan young men more often choose to postpone strong relationship commitments, such as marriage. Our data did not allow us to include other types of indicators in our analyses, but more research is clearly needed to understand the dynamics of the union formation patterns of second-generation men and women of different origins.

Our work supports the assumption that the diffusion of ideas and innovations results in changes over cohorts. However, other factors, that we were unable to include in our study given the available data, may have affected the observed patterns. First, previous research has shown that members of the second generation in the Netherlands often marry a partner born in their parents’ country of origin (Huschek et al., 2012). Marrying a partner from abroad has, however, become more difficult over time due to the introduction of stricter income and age requirements in 2004 for people who want to move to the Netherlands. These restrictions may have resulted in lower marriage rates across birth cohorts (WODC/INDIAC, 2009). Another way in which partner choice could be related to the postponement of marriage would be an increase in exogamous marriages as these tend to take place at older ages (Soehl & Yahirun, 2011). Moreover, preferences for second-generation partners or first-generation partners could play a role as well. How partner choice and changes therein are related to changes in marriage timing is an important avenue for future research in order to better understand partnering dynamics.

Second, we observe an increase in marriage ages in Turkey and Morocco as well (Assaad & Krafft, 2015; HUIPS, 2014). While it is possible that the marriage behaviour of the second generation in the Netherlands is being influenced through ties with the country of origin, it may also be the case that delaying marriage has become more common in general.

Although our study clearly shows the role of cohort succession among the second generation, it also has a number of limitations. First, although our full population register data provide unique opportunities for studying cohort succession, they are more limited in covering other variables that previous studies have found to be relevant for the timing of marriage, like religiosity and parental education. Overall, more religious people tend to marry at younger ages (Rendon et al., 2014). It is therefore important for future research to include religiosity when studying the second generation (a characteristic not available in the population registers in the Netherlands). Moreover, a recent study by Mooyaart and Liefbroer (2016) showed that the influence of the mother’s education on the timing of union formation changes across cohorts. How this process works and may be relevant for the second generation remains unclear. Second, as the registers include information on each person’s level of education only from 2006 onward, we used the highest level of completed education at the end of our observation period as an indicator of each individual’s final educational level. In the Netherlands, the stacking of education is relatively common, especially among migrant groups. Therefore, some of the individuals in our study population might have finished a higher level of education after the end of our observation period, which would lead to an underestimation of individuals with medium–high and high levels of education in the younger cohorts. Future research would benefit from including educational level measured prior to early adulthood in order to prevent causality issues. Third, as we mentioned before, a pathway for future research would be to follow birth cohorts for a longer time period in order to determine whether the postponement of marriage eventually leads to lower total marriage rates. If so, this would indicate convergence of the union formation patterns of the second generation with the majority population, not only at the younger ages, but overall.

In sum, this study provides a detailed analysis of changes in the timing of marriage across different birth cohorts of second-generation young adults. The findings confirm expectations about postponement of marriage based on diffusion theories and point to the need to account for cohort effects in future studies. Whether these changes indicate the unfolding of the second demographic transition (Lesthaeghe, 2010) or a second-generation specific pattern needs to be studied. Although the Netherlands provides a relevant case study due to its large second-generation population and the availability of high-quality register data, future research would benefit from including other national contexts and migrant backgrounds. The differences in the union formation patterns of the country of residence and of the parents’ country of origin are likely to influence the diffusion process among the second generation, and the changes in these patterns across birth cohorts. In any case, our study shows that without taking the cohort change in timing of marriage into account, studies comparing the second generation with the majority population might overestimate the differences between those with and without migrant origin.

## Appendix

See Figs. 3, 4, 5, 6, 7 and 8 and Table 5.

**Table 5:** Results of discrete-time logistic regression models including interaction terms between birth cohorts and origin for men and women

	Men		Women	
	OR (SE)	95% CI	OR (SE)	95% CI
Age	2.220 (0.047)	2.130–2.314	1.881 (0.019)	1.844–1.919
Age squared	0.960 (0.002)	0.957–0.963	0.959 (0.001)	0.957–0.960
Birth cohort (ref = 1980)				
1981	0.945 (0.055)	0.843–1.059	0.928 (0.036)	0.859–1.001
1982	1.051 (0.060)	0.939–1.177	0.911 (0.036)	0.844–0.984
1983	0.942 (0.056)	0.838–1.058	0.902 (0.036)	0.835–0.975
1984	0.979 (0.057)	0.873–1.097	0.887 (0.035)	0.820–0.959
1985	1.022 (0.059)	0.914–1.144	0.806 (0.033)	0.744–0.873
1986	0.955 (0.055)	0.852–1.071	0.831 (0.034)	0.768–0.899
1987	0.864 (0.052)	0.768–0.972	0.833 (0.034)	0.770–0.902
1988	0.913 (0.052)	0.812–1.025	0.733 (0.031)	0.676–0.796
1989	0.757 (0.047)	0.670–0.855	0.705 (0.030)	0.649–0.767
1990	0.743 (0.046)	0.658–0.839	0.651 (0.028)	0.599–0.709
Origin (ref = Dutch)				
Moroccan	2.660 (0.208)	2.283–3.099	3.386 (0.184)	3.044–3.766
Turkish	6.944 (0.407)	6.191–7.788	5.856 (0.278)	5.336–6.426
1981*M	0.768 (0.087)	0.615–0.958	0.980 (0.075)	0.843–1.138
1981*T	0.892 (0.075)	0.757–1.051	1.033 (0.068)	0.908–1.175
1982*M	0.788 (0.085)	0.637–0.974	1.066 (0.080)	0.920–1.234
1982*T	0.733 (0.061)	0.622–0.864	0.948 (0.064)	0.829–1.082
1983*M	0.929 (0.100)	0.752–1.147	1.069 (0.079)	0.924–1.236
1983*T	0.750 (0.064)	0.635–0.886	0.927 (0.062)	0.813–1.058
1984*M	0.916 (0.098)	0.743–1.129	0.953 (0.071)	0.824–1.103
1984*T	0.657 (0.055)	0.556–0.775	0.870 (0.058)	0.763–0.993
1985*M	0.888 (0.093)	0.723–1.090	1.142 (0.084)	0.988–1.320
1985*T	0.623 (0.052)	0.528–0.734	0.878 (0.060)	0.768–1.003
1986*M	0.890 (0.094)	0.725–1.094	1.065 (0.077)	0.923–0.837
1986*T	0.579 (0.050)	0.506–0.705	0.735 (0.049)	0.644–0.837
1987*M	1.059 (0.111)	0.863–1.300	0.986 (0.072)	0.854–1.137
1987*T	0.622 (0.053)	0.527–0.734	0.717 (0.047)	0.631–0.815
1988*M	0.884 (0.092)	0.721–1.084	1.068 (0.078)	0.925–1.232
1988*T	0.521 (0.044)	0.442–0.614	0.739 (0.048)	0.650–0.841
1989*M	1.005 (0.106)	0.817–1.236	1.051 (0.076)	0.911–1.212
1989*T	0.641 (0.054)	0.543–0.757	0.736 (0.048)	0.648–0.837
1990*M	1.040 (0.108)	0.849–1.274	0.995 (0.073)	0.862–1.148
1990*T	0.591 (0.050)	0.501–0.697	0.729 (0.048)	0.642–0.829
Employed mother age 15	0.767 (0.014)	0.739–0.796	0.811 (0.11)	0.789–0.833
Educational level (ref = Middle)				
Low	0.686 (0.016)	0.656–0.717	0.961 (0.019)	0.925–0.999
Medium–high	1.051 (0.026)	1.002–1.103	0.780 (0.013)	0.755–0.806
High	0.851 (0.028)	0.798–0.907	0.479 (0.012)	0.457–0.503
Enrolled in education	0.336 (0.009)	0.320–0.354	0.476 (0.007)	0.462–0.490
Constant	0.001 (0.000)	0.001–0.001	0.005 (0.000)	0.005–0.006
*N* Individuals	124,974		119,813	
*N* Person-years	1,216,277		1,9099,983	

*OR* odds ratio; *SE* standard error; 95% *CI* 95 percent confidence interval

M = Moroccan second generation, T = Turkish second generation

## Footnotes

1 For those with a Dutch origin, control variables specific to the second generation were not used in the imputation model.

2 Analyses excluding individuals with missing values on the highest level of completed education and employment of the mother produced highly similar results.

3 The number of individuals with one foreign-born parent in our study population and among these groups in general is small (5–7 per cent). Results from additional analyses excluding these individuals resulted in overall similar results (detailed analyses available upon request from the first author). Since we found no bias, we decided to continue modelling including all individuals.

4 We took out the control variables measuring ties to the country of origin, number of foreign-born parents and percentage of co-ethnics in the neighbourhood as these do not apply to the majority population.
